# Retrospective analysis of 98 cases of maxillary sinus squamous cell carcinoma and therapeutic exploration

**DOI:** 10.1186/s12957-020-01862-3

**Published:** 2020-05-06

**Authors:** Yu Wang, Rong Yang, Minghui Zhao, Wenyu Guo, Lun Zhang, Wenchao Zhang, Xudong Wang

**Affiliations:** 1 Department of Maxillofacial and Otorhinolaryngology Oncology, Tianjin Medical University Cancer Institute and Hospital, Key Laboratory of Cancer Prevention and Therapy, Tianjin Cancer Institute, National Clinical Research Center of Cancer, Tianjin, 300060 China; 2 Molecular Diagnostic Laboratory of Cancer Center, Shanghai General Hospital, Shanghai Jiao Tong University School of Medicine, Shanghai, 201620 China

**Keywords:** Maxillary sinus squamous cell carcinoma, Surgery, Comprehensive treatment, Neoadjuvant chemoradiotherapy, Adjuvant chemotherapy, Adjuvant radiotherapy

## Abstract

**Background:**

Maxillary sinus squamous cell carcinoma (MSSCC) is a relatively rare head and neck cancer with poorly defined prognosis, and the present study aimed to investigate the outcomes for MSSCC according to different treatments.

**Methods:**

Tianjin Medical University Cancer Institute and Hospital pathology database was reviewed from 2007 to 2017, and 98 patients with pathologically confirmed MSSCC were enrolled. Retrospective analysis and follow-up were performed for each patient. Multivariate analysis of prognostic factors was performed using Cox’s regression model.

**Results:**

For all the 98 cases of MSSCC, the 5-year overall survival (OS) and disease-free survival (DFS) rates were 31.0% and 29.3%, respectively. Among 98 patient, 33 patients were treated with systemic treatment (NON-SUR), 19 patients underwent neoadjuvant chemotherapy and/or radiotherapy followed by surgery (CT/RT+SUR), 38 patients received surgery followed by chemotherapy and/or radiotherapy (SUR+RT/CT), and 8 patients were performed surgery alone (SUR).The OS rate for each group was 27.3%, 57.9%, 30.6% and 37.5%, respectively, while the DFS was 21.2%, 36.8%, 31.6% and 25.0%, respectively. The OS rate of CT/RT+SUR was significantly higher than that of NON-SUR and SUR+CT/RT groups (*P* < 0.05). Multivariate analysis revealed that smoking, low differentiation, and advanced T stage were independent risk factors for OS, while low differentiation and advanced N stage for DFS.

**Conclusions:**

Surgery-based treatment is still the first-line therapeutic strategy for MSSCC. And neoadjuvant chemoradiotherapy followed by surgery is highly recommended for MSSCC patients, especially those with advanced tumors or requesting high quality of life.

## Introduction

Maxillary sinus carcinoma (MSC) is a relatively rare neoplasm with a poorly defined prognosis [1]. Maxillary sinus squamous cell carcinoma (MSSCC) is the most common pathological type in MSC, and nearly 80% of MSSCCs are diagnosed at advanced stages due to a lack of typical symptoms [2, 3]. Although the mainstay of treatment for MSSCC has been well developed recently, the 5-year overall survival (OS) rate remains unsatisfactory [4].

It is generally accepted that surgery-based treatment remains the first-line therapeutic strategy for MSSCC [5–8], according to the National Comprehensive Cancer Network (NCCN) recommendation [8]. And comprehensive treatment involving surgery was observed to have a better curative effect than surgery alone [9–11]. However, controversial issues still exist that whether neoadjuvant chemotherapy and/or radiotherapy benefit patient prognosis better, comparing with post-operation adjuvant chemotherapy and/or radiation. Based on these, the objective of this retrospective study was to explore the survival outcomes of MSSCC patients according to different treatments.

## Material and methods

### Patients

We performed a retrospective review of pathological databases from 2007 to 2017 at Tianjin Medical University Cancer Institute and Hospital, and 98 patients with pathologically confirmed MSSCC were enrolled. Patients were classified according to the 8th edition of the American Joint Committee on Cancer (AJCC) TNM staging system [12]. The current study was approved by the Institutional Review Board (IRB) of the Tianjin Medical University Cancer Institute and Hospital, and conducted in accordance with the Helsinki Declaration.

### Treatment selection criteria

According to the NCCN guidelines (Head and Neck Cancers), surgical resection was recommended for patients with resectable tumors and willing to undergo surgery (no positive margins or extra-lymph node extension) (SUR). Patients with positive margins and extra-lymph node extension are recommended for adjuvant radiotherapy or systemic treatment (SUR+CT/RT). For patients with T4b stage or distance metastasis, radiotherapy and/or chemotherapy will be performed (NON-SUR). For patients downstaged from unresectable to resectable after radiotherapy and/or systemic treatment, operation is recommended (CT/RT+SUR). And for patients unwilling to undergo surgery or requesting high quality of life but with resectable tumors, neoadjuvant therapy followed by surgery is still highly recommended (CT/RT+SUR). Cervical lymph node radical dissection will be performed for N+ patients, and appropriate flaps will be used for maxillofacial reconstruction.

### Neoadjuvant or adjuvant therapy

Chemotherapy (CT) was performed with a TPF regimen, including docetaxel (75 mg/m^2^ day 1), cisplatin (75 mg/m^2^ day 2–3), and 5-FU (750 mg/m^2^ day 2–3), 3 weeks/cycle. Neoadjuvant chemotherapy was defined as chemotherapy starting 2 months before surgery with 2 or 3 cycles. Adjuvant chemotherapy was performed within 2 months after surgery with 2 or 3 cycles.

Radiotherapy (RT) was divided into radical RT and adjuvant RT. The target area ranged from 1.5 to 2 cm outside the boundary of the clinical lesion. Radical RT was performed for patients without surgery at a mean dose of 66 Gy for the primary lesion and 44–50 Gy for the suspected subclinical spread area, 5 days a week for a total of 6–7 weeks. Adjuvant RT was defined as RT beginning within 1 month after surgery, of which the primary lesion dose was reduced to 60 Gy. Preoperative radiotherapy was performed with the same dose as that used for adjuvant radiotherapy within 3 months before the operation. Concurrent chemoradiotherapy (CCRT) was defined as CT followed by RT once a week. Continuous chemoradiotherapy (CRT) was defined as CT after RT.

### Follow-up

Patients were reviewed every 3 months within 1 year after the end of treatment, every 6 months within 5 years, and once a year after 5 years. Nasopharyngoscopy, computed tomography (CT), and magnetic resonance imaging (MRI) were performed during the follow-up for evaluation. OS was calculated as the period of time from the date of diagnosis to the date of death from any cause or the date of the last follow-up. Disease-free survival (DFS) was defined as the period of time from the date of diagnosis to the date of recurrence or the date of death due to cancer progression.

### Statistical analysis

Data analysis was performed using SPSS 25.0 (IBM Analytics, USA). Categorical variables were compared using the chi-square test. Fisher’s exact test was used to analyze samples less than 5, and continuous variables were analyzed with Mann-Whitney *H* test. Kaplan-Meier and log-rank tests were performed to evaluate OS and DFS rate. Cox regression models were used to estimate the association between treatment and survival. *P* values < 0.05 were considered statistically significant.

## Results

### Clinical characteristics of MSSCC patients

The present study involved 98 patients with MSSCC consisted of 75 males and 23 females, with a median age of 57.5 years (range 22–85). With Ohngren’s line as the boundary, we determined that 55 tumors invaded the upper structures and 43 invaded the lower. And all the clinical characteristics of 98 MSSCC patients are summarized in Table 1.

**Table 1:** Clinical features of and univariate analysis of MSSCC

Variable	*n* (%)	Median OS	OS	*P* value	DFS	*P* value
Total	98 (100)	23 (3–120)	35.70%		28.60%	
Sex				0.904		0.464
Female	23 (23.5)	26 (3–98)	39.10%		34.80%	
Male	75 (76.5)	17 (3–120)	34.70%		26.70%	
Age				0.329		0.448
≤55	38 (38.8)	32 (3–120)	36.80%		28.30%	
>55	60 (61.2)	17.5 (3–120)	35.00%		27.90%	
Smoking				0.038*		0.342
No	31 (31.6)	33 (5–120)	51.60%		32.30%	
Yes	67 (68.4)	19 (3–98)	28.40%		26.90%	
Alcohol				0.209		0.464
No	41 (41.8)	24 (6–120)	46.30%		34.10%	
Yes	57 (58.2)	25 (3–98)	28.10%		24.60%	
Differentiation				< 0.001*		0.001*
Highly	32 (32.7)	42.5 (8–120)	46.90%		37.50%	
Medium	33 (33.7)	27 (6–85)	33.30%		24.20%	
Low	33 (33.7)	15 (3–89)	27.30%		24.20%	
Invasion direction				0.003*		< 0.001*
Up	43 (56.1)	15 (3–89)	45.50%		38.20%	
Down	55 (43.9)	32 (3–120)	23.30%		16.30%	
T stage				0.002*		0.057
T1	3 (3.1)	67 (31–120)	100.00%		75.00%	
T2	14 (14.3)	39.5 (9–95)	57.10%		42.90%	
T3	37 (37.8)	27 (6–120)	37.80%		27.80%	
T4	44 (44.9)	18.5 (3–76)	22.70%		20.50%	
N stage				0.002*		0.022*
N0	75 (76.5)	32 (3–120)	40.00%		29.30%	
N1	4 (4.1)	12 (5–17)	0%		0%	
N2	19 (19.4)	13 (3–89)	26.30%		31.60%	
M stage				0.531		0.108
M0	95 (95.9)	25 (3–120)	35.80%		29.50%	
M1	3 (4.1)	15.5 (3–32)	33.30%		0.00%	
Clinical stage				0.015*		0.034*
I	3 (3.1)	67 (31–120)	100.00%		100.00%	
II	9 (9.2)	54 (10–95)	50.00%		40.00%	
III	28 (28.6)	29.5 (8–98)	42.90%		28.60%	
IV	58 (59.2)	19 (3–120)	26.30%		22.80%	

*Statistical significance

### Therapeutic procedure

In the current study, a total of 65 patients with MSSCC underwent surgery, including maxillary radical resection (*n* = 13), total maxillectomy (*n* = 23), subtotal maxillary resection (*n* = 9), and endoscopic surgical resection (*n* = 15). Cervical lymph node dissection was performed for patients with lymph node metastasis (*n* = 15). Orbital exenteration was performed on 3 patients. Musculocutaneous flaps were used in 5 patients, and skin grafts were used in 11 patients for maxillofacial reconstruction.

Among 98 MSSCC patients, 33 in the NON-SUR group, 19 in the CT/RT+SUR group, 38 in the SUR+RT/CT group, 8 in the SUR group. In detail, neoadjuvant chemotherapy and/or radiotherapy was administered in 19 patients (CT/RT+SUR) [CT+SUR, *n* = 2; RT+SUR, *n* = 7; CRT+SUR, *n* = 10]. Thirty-three patients were treated with nonsurgical treatment (NON-SUR) [RT, *n* = 2; CT, *n* = 5; CCRT, *n* = 13; CRT, *n* = 13]. And a total of 38 patients received operation followed by adjuvant chemotherapy and/or radiotherapy (SUR+RT/CT) [SUR+CCRT, *n* = 12; SUR+CRT, *n* = 7; SUR+CT, *n* = 5; SUR+RT, *n* = 14].

### Therapeutic outcomes of MSSCC patients

**Fig. 1:**
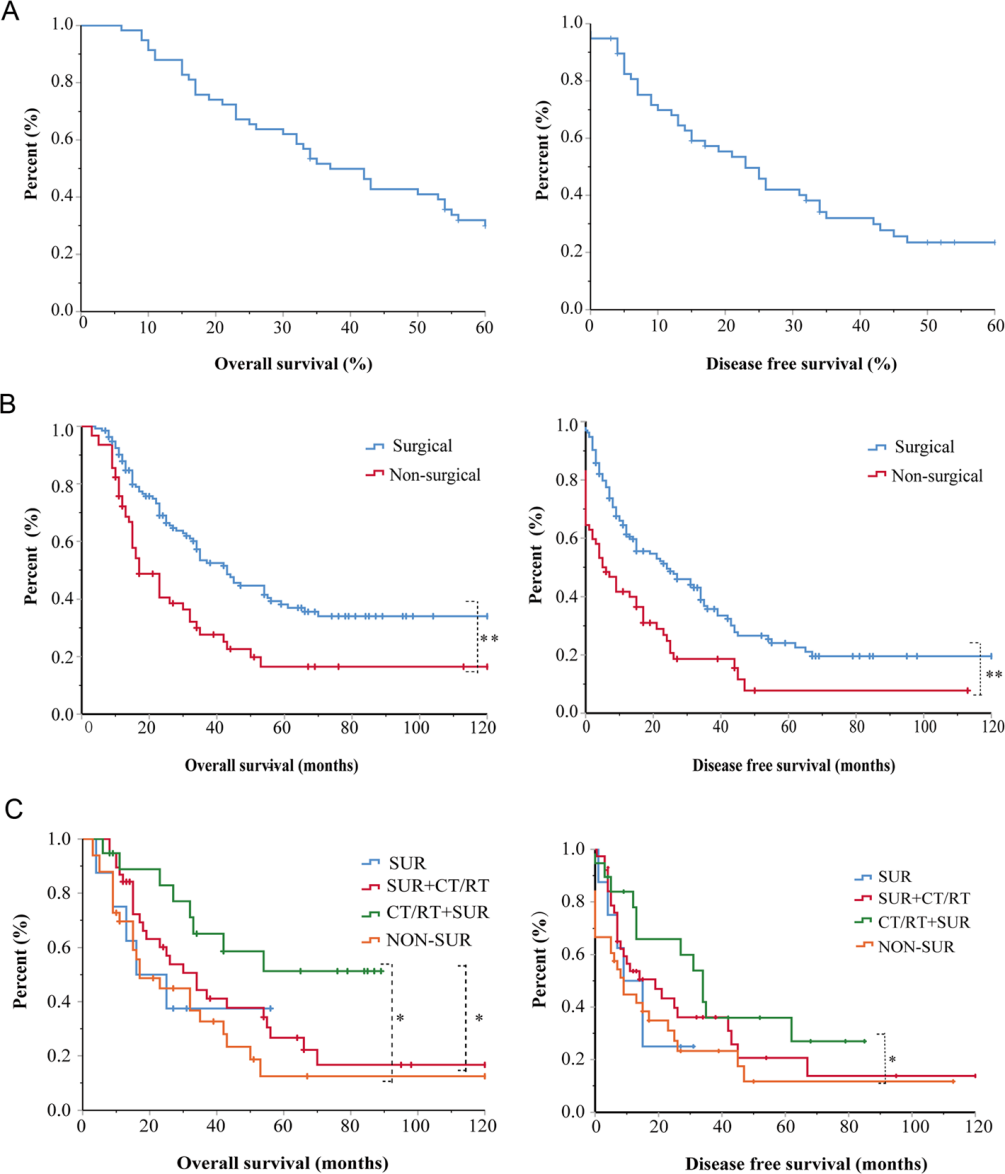
Clinical characteristics of MSSCC patients. **a** The 5-year OS and DFS curve for MSSCC patients. **b** OS curves and DFS curves for MSSCC patients after surgical and nonsurgical treatment. **c** OS curves and DFS curves for MSSCC patients after different treatments. *P < 0.05, **P < 0.001

Among the 98 MSSCC patients, the median follow-up time was 36 months (range 12–120 months). The 5-year OS and DFS rates were 31.0% and 29.3%, respectively (Fig. 1a). Sixty-five patients underwent surgery, and 33 patients were treated with nonsurgical treatment. The OS rate was 40.6% in the surgical group and 26.5% in the nonsurgical group, and the DFS rates were 32.8% and 20.6%, respectively. Both the OS and DFS rates of the surgical group were significantly better than those of the nonsurgical group (*P* < 0.001) (Fig. 1b).

Mountains of evidence revealed that comprehensive treatment involving surgery may have a better curative effect than surgery alone [1, 9–11]. Similarly, in the present study, 98 patients with MSSCC were divided into four groups: SUR (*n* = 8), SUR+CT/RT (*n* = 38), CT/RT+SUR (*n* = 19), and NON-SUR (*n* = 33). The OS rates were 37.5%, 31.6%, 57.9%, and 27.3%, respectively, while the DFS rates were 25.0%, 31.6%, 36.8%, and 28.4%, respectively. OS was significantly better in the CT/RT+SUR group than that in the SUR+CT/RT or NON-SUR group (*P* < 0.05). However, there was no significant difference in DFS among these three groups (Fig. 1c). MSSCC patients benefited more from chemotherapy and/or radiotherapy followed by surgery (*P* = 0.113, hazard ratio [HR] = 0.135) and surgery followed by chemotherapy and/or radiotherapy (*P* = 0.751, HR = 0.736) than from surgery alone. Furthermore, the HR index of CR/RT+SUR group was much lower than that of SUR+CT/RT group, which partially indicates that chemotherapy and/or radiotherapy followed by surgery is more beneficial than surgery followed by chemotherapy and/or radiotherapy for MSSCC (Table 2).

**Table 2:** Multivariate analyses of MSSCC

Characteristic	OS	*P* Value	DFS	*P* Value
	HR (95% CI)		HR (95% CI)	
Smoking				
No	[Reference] 1		[Reference] 1	
Yes	2.098(1.140–3.862)	0.017*	NA	0.377
Differentiation				
Highly	[Reference] 1		[Reference] 1	
Medium	1.784 (0.936–3.398)	0.078	1.648(0.909–2.988)	0.1
Low	3.473 (1.793–6.725)	< 0.001*	2.970(1.597–5.524)	0.001*
T stage				
T1	[Reference] 1		[Reference] 1	
T2	0.711 (0.277–1.824)	0.479	NA	0.336
T3	0.439 (0.146–1.325)	0.144	NA	0.928
T4	2.454 (1.099–5.477)	0.028*	NA	0.059
N stage				
N0	[Reference] 1		[Reference] 1	
N1	NA	0.192	3.400(1.068–10.828)	0.038*
N2	NA	0.148	1.742(0.738–4.116)	0.205
M stage				
M0	[Reference] 1		[Reference] 1	
M1	NA	0.248	NA	0.046
Clinical stage				
I	[Reference] 1		[Reference] 1	
II	NA	0.346	NA	0.883
III	NA	0.267	NA	0.798
IV	NA	0.663	NA	0.167
Treatment				
SUR	[Reference] 1		[Reference] 1	
SUR+CT/RT	0.853 (0.319–2.270)	0.751	0.852(0.336–2.161)	0.736
CT/RT+SUR	0.369 (0.107–1.267)	0.113	0.510(0.167–1.551)	0.235
NON-SUR	1.054 (0.381–2.919)	0.919	0.910(0.315–2.362)	0.846

*HR* hazard ratio, *CI* confidence interval, *NA* not applicable

*Statistical significance

### Prognostic factors of MSSCC

**Fig. 2:**
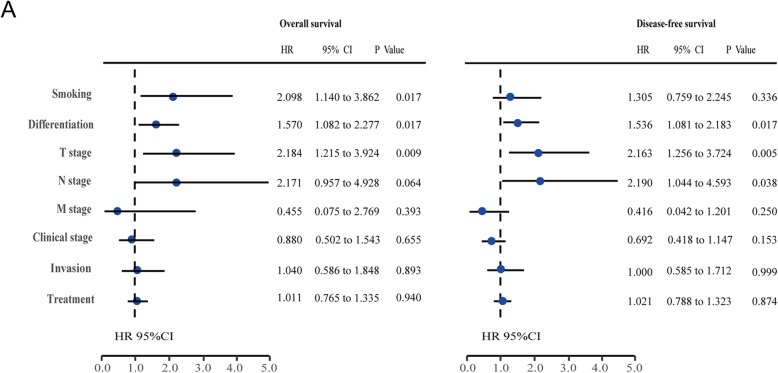
Prognostic factors of MSSCC. **a** Multivariate Cox regression analyses of MSSCC

The results of the univariate analysis showed that smoking, tumor differentiation, TNM stage, clinical stage, and tumor invasion direction were prognostic factors for OS (*P* < 0.05), while differentiation, T stage, N stage, and clinical stage were prognostic factors for DFS (*P* < 0.05) (Table 1). The multivariate analysis revealed that smoking (HR = 2.098), advanced T stage (HR = 2.184), and low differentiation (HR = 1.570) were independent prognostic factors for OS (*P* < 0.05), and low differentiation (HR = 1.536), advanced T stage (HR = 2.163), and N stage (HR = 2.190) were independent prognostic factors for DFS (*P* < 0.05) (Table 2 and Fig. 2a).

## Discussion

MSSCC is a highly aggressive human cancer with a relatively poor prognosis [13], accounting for approximately 80% of malignant paranasal sinus tumors [14]. As mentioned in the literature, the OS outcomes are still unsatisfactory despite the development of diagnosis and therapeutic strategies [15]. The results of this study showed that the 5-year OS and DFS rates of MSSCC patients were 31.0% and 29.3%, respectively. According to the guidelines published by the NCCN, surgery followed by adjuvant chemoradiotherapy is highly recommended as the preferred method for resectable MSSCC (T1-T4a) [8]. In addition, surgical resection followed by adjuvant radiotherapy is widely performed during the treatment of MSSCC [4, 5, 7, 16, 17]. Consistently, the present study also suggested that surgery-based treatment is more beneficial than systemic therapy. However, Park et al. reported a relatively high local recurrence rate after SUR+RT treatment [18]. Furthermore, Kuo and colleagues demonstrated that neoadjuvant treatment was associated with improved OS in MSSCC patients [19]. Thus, whether neoadjuvant or postoperative adjuvant chemoradiotherapy benefits patients is still controversial.

Chemoradiotherapy was confirmed to play an important part in the treatment of MSSCC, especially for tumors with positive margins or high-risk features [20, 21]. Mountains of evidence have shown that postoperative chemotherapy and/or radiotherapy are associated with better survival outcomes of MSSCC patients compared with surgery alone [9, 16, 21]. However, studies that focused on comparisons between upfront surgery and neoadjuvant chemotherapy and/or radiotherapy followed by surgery in patients with MSSCC showed different results [19]. For advanced MSSCC, neoadjuvant chemotherapy or radiotherapy is also viewed globally to downstage tumors for surgical resection [22, 23]. In our study, the OS rates of the SUR, SUR+CT/RT, and CT/RT+SUR groups were 37.5%, 31.6%, and 57.9%, respectively. The HRs of the CT/RT+SUR and SUR+RT/CT groups were 0.751 and 0.356, respectively (relative to the SUR group). These results demonstrated that chemotherapy and/or radiotherapy followed by surgery was associated with favorable OS rates of MSSCC patients compared to surgery followed by chemotherapy and/or radiotherapy or surgery alone. Considering the advantages of preoperative treatment, neoadjuvant chemotherapy and/or radiotherapy followed by surgery might be more beneficial and is highly recommended for MSSCC patients. Besides, the multiple regression analysis revealed that smoking and advanced T stage were independent risk factors for survival, suggesting smoking cessation, and early diagnosis of MSSCC. When patients have chronic nasal congestion or bloody nose, a CT scan or an MRI examination is recommended, and a pathological biopsy should be performed for diagnosis when necessary.

Nevertheless, this study was limited by the relatively small number of samples in a single cancer center. Consequently, larger retrospective analysis and further multi-institutional clinical trials are required for a much more detailed analysis of this rare malignant tumor.

## Conclusions

Surgery-based comprehensive treatment is still the first-line approach for MSSCC. Limited by the nature of retrospective studies, it is too early to draw conclusions that neoadjuvant radiotherapy or chemotherapy could improve the overall survival of MSSCC. Even so, neoadjuvant radiotherapy and/or chemotherapy is believed to create surgical opportunities for patients with unresectable tumors and bring about higher quality of life to some extent. Thus, neoadjuvant chemoradiotherapy followed by surgery is still recommended for MSSCC patients, especially those with advanced tumors or requesting high quality of life.

## Data Availability

The patient data will not be shared. All of the patient data was collected from Tianjin Medical University Cancer Institute and Hospital surgical and pathological databases. All patients provided written consent for storage of their information in the hospital database only.

## Abbreviations

MSC: Maxillary sinus carcinoma
MSSCC: Maxillary sinus squamous cell carcinoma
